# Heterologous expression and structural characterization of polyamide 4-degrading enzyme from a soil bacterium

**DOI:** 10.3389/fmicb.2026.1811100

**Published:** 2026-06-02

**Authors:** Yusuke Saito, Shunko Sato, Yurika Sasanami, Tetsuro Yamashita, Miwa Yamada

**Affiliations:** 1 Department of Bioresources Science, The United Graduate School of Agricultural Sciences, Iwate University, Morioka, Iwate, Japan; 2 Department of Life Sciences, Studies in Molecular Biology and Biochemistry, Iwate University, Morioka, Iwate, Japan; 3 Agri-Innovation Center, Iwate University, Morioka, Iwate, Japan; 4 Center for Sustainable Materials and Interfacial Science (CSMIS), Iwate University, Morioka, Iwate, Japan

**Keywords:** biodegradation, bioplastic, *Brevibacillus choshinensis* HPD31-SP3, nylon 4, *Pseudoxanthomonas* sp.

## Abstract

Polyamide 4 (PA4) is a bio-based plastic with thermal stability, excellent mechanical properties, and good biodegradability in various environments. To understand the biodegradation of PA4 under natural environments, PA4-degrading microorganisms and enzymes have been investigated. Although our previous research identified the amino acid sequence and predicted the three-dimensional (3D) structure of a PA4-degrading enzyme from a marine environment (Nyl4A*_pa_*), those of an enzyme from terrestrial environments have remained unidentified. In this study, we identified the PA4-degrading enzyme gene (*nyl4A_px_*) from the PA4-degrading soil bacterium *Pseudoxanthomonas* sp. TN-N1. In addition, *nyl4A_px_* was successfully expressed in *Escherichia coli* BL21(DE3) and *Brevibacillus choshinensis* HPD31-SP3. The PA4-degrading activity of the enzyme secreted by recombinant *B. choshinensis* HPD31-SP3 reached 68.8 Δ655 nm/h/100 mL broth, representing a 2.4-fold increase compared with that produced by recombinant *E. coli* BL21(DE3). Based on a homology search using the amino acid sequence and predicted 3D structure of the enzyme, Nyl4A*_px_* was predicted to be composed of a substrate-binding domain, a middle domain, and a catalytic domain. Among these domains, the substrate-binding and catalytic domains of Nyl4A*_px_* are sequentially and structurally similar to those of Nyl4A*_pa_*. Furthermore, putative homologs of Nyl4A*_px_* and Nyl4A*_pa_* were found in marine-associated environmental metagenomes through BLAST searches. To our knowledge, this is the first report describing the structural properties of a PA4-degrading enzyme from a soil bacterium.

## 1 Introduction

Environmental pollution caused by large amounts of plastic waste has become a serious global issue. The amount of plastic waste has continued to increase every year, reaching approximately 335 million tons in 2016. If the current trend continues, this number is estimated to double by 2050 (Macheca et al., 2024). Most plastic products are eventually released into the environment, where they accumulate and cause severe damage to ecosystems (Chae and An, 2018). To address this issue, biodegradable plastics are being developed as substitutes for conventional plastics, which are resistant to degradation in natural environments.

Polyamide 4 (PA4) is a bio-based plastic that exhibits thermal stability and mechanical properties comparable to those of polyamide 6, a material widely used in textiles and industrial components (Fukuda and Sasanuma, 2018). PA4 is polymerized from 2-pyrrolidone, a cyclic form of gamma-aminobutyric acid (GABA). GABA is produced by the *α*-decarboxylation of glutamic acid catalyzed by glutamic acid decarboxylase (Yamano et al., 2012; Sarasa et al., 2020). Recombinant *Escherichia coli* capable of one-pot conversion of 2-pyrrolidone from bio-based glutamic acid has been developed, demonstrating that the PA4 monomer can be produced from biomass through a completely biotechnological process (Zhang et al., 2016). Furthermore, unlike conventional polyamides that exhibit low biodegradability in the environment, PA4 is highly biodegradable in natural environments, including soil (Hashimoto et al., 1994; Tachibana et al., 2010; Wang et al., 2023), activated sludge (Yamano et al., 2008), and the ocean (Tachibana et al., 2013; Yamano et al., 2019). In particular, the marine biodegradability of PA4 is advantageous and contributes to addressing marine plastic waste, as few biodegradable plastics possess this property (Suzuki et al., 2020). Due to these favorable properties, PA4 is expected to replace conventional plastics.

Understanding the microbial and enzymatic mechanisms underlying the degradation of biodegradable plastics in the environment will help to predict their ecological impact. Genetic information on PA4-degrading microorganisms and enzymes identified in the environment can be used to predict their natural distribution based on metagenomic data under various environmental conditions, thereby enabling the simulation of PA4 degradation levels in nature. Furthermore, predicting the impact of PA4 degradation products on ecosystems is possible. Therefore, it is necessary to identify a large number of PA4-degrading microorganisms and enzymes and accumulate information on them; however, reports on PA4-degrading microorganisms identified in the environment are currently limited (Tachibana et al., 2010; Wang et al., 2023; Yamano et al., 2008; Yamano et al., 2019; Sasanami et al., 2022; Saito et al., 2023; Saito et al., 2024), and only two types of PA4-degrading enzymes have been reported thus far (Sasanami et al., 2022; Saito et al., 2023).

Recently, we successfully identified extracellular PA4-degrading enzymes, which hydrolyze PA4 at amide bonds to produce GABA oligomers, from *Pseudoxanthomonas* sp. TN-N1 and *Pseudoalteromonas* sp. Y-5 isolated from terrestrial and marine environments, respectively (Sasanami et al., 2022; Saito et al., 2023). We also identified the PA4-degrading enzyme gene (Nyl4A*_pa_*, GenBank Accession No. LC765678.1) from the marine PA4-degrading bacterium *Pseudoalteromonas* sp. Y-5 and reported the structural properties of Nyl4A*_pa_* for the first time (Saito et al., 2023). BLAST search suggested that the C-terminal region of Nyl4A*_pa_* belongs to the AmpC superfamily; however, most of the homologous sequences were annotated as hypothetical proteins. Nyl4A*_pa_* was estimated to be a homodimer composed of 75 kDa subunits. Structural prediction using AlphaFold2 indicated that the Nyl4A*_pa_* subunit consists of a substrate-binding domain and a catalytic domain. The catalytic domain was predicted to be structurally similar to some β-lactamase family proteins and a 6-aminohexanoate-dimer hydrolase (Negoro et al., 2007). However, Nyl4A*_pa_* did not exhibit β-lactamase activity or the ability to degrade polyamide 6 or 6-aminohexanoate-dimer, suggesting that Nyl4A*_pa_* belonged to a novel type of protein. In contrast, the PA4-degrading enzyme identified from *Pseudoxanthomonas* sp. TN-N1 was estimated to be a monomer or dimer composed of 90 kDa subunits, suggesting that its subunit structure differs from that of the marine-derived Nyl4A*_pa_.* In addition, although the terrestrial-derived PA4-degrading enzyme can hydrolyze the amide bonds of PA4, no protease activity was detected. Therefore, this enzyme could be a novel enzyme. However, its amino acid sequence and three-dimensional (3D) structure have not yet been revealed.

Herein, we report the amino acid sequence and structural properties of the PA4-degrading enzyme from *Pseudoxanthomonas* sp. TN-N1 (Nyl4A*_px_*) isolated from soil. We identified the PA4-degrading enzyme gene from *Pseudoxanthomonas* sp. TN-N1 and performed gene cloning. The PA4-degrading enzyme gene was heterologously expressed in *Escherichia coli* BL21(DE3) or *Brevibacillus choshinensis* HPD31-SP3. In addition, we predicted the 3D structure of Nyl4A*_px_* based on the identified amino acid sequence using AlphaFold2 and compared its amino acid sequence and predicted 3D structure with those of previously reported Nyl4A*_pa_*.

## 2 Materials and methods

### 2.1 Bacterial strains, culture medium, and plasmids

*Escherichia coli* JM109 was used as the host strain for plasmid construction. *E. coli* BL21(DE3) and *B. choshinensis* HPD31-SP3 were used as host strains for protein expression. *E. coli* strains were cultured in Luria-Bertani (LB) medium [pH 7.0; components (/L): Bacto tryptone, 10 g; yeast extract, 5 g; NaCl, 5 g]. *B. choshinensis* HPD31-SP3 was cultured in TM medium [pH 7.0; components (/L): phytone peptone, 10 g; 35% ehrlich bonito extract, 5.8 g; yeast extract, 2 g; glucose, 10 g; FeSO_4_·7H_2_O, 10 mg; MnSO_4_·4H_2_O, 10 mg; ZnSO_4_·7H_2_O, 1 mg]. *B. choshinensis* HPD31-SP3 harboring plasmids was selected on MT plates [pH 7.0; components (/L): phytone peptone, 10 g; 35% ehrlich bonito extract, 5.8 g; yeast extract, 2 g; glucose, 10 g; FeSO_4_·7H_2_O, 10 mg; MnSO_4_·4H_2_O, 10 mg; ZnSO_4_·7H_2_O, 1 mg; MgCl_2_·6H_2_O, 4.1 g; agar, 15 g]. pColdIV and pNCMO2 were used as cloning vectors for *E. coli* and *B. choshinensis*, respectively.

### 2.2 Cloning of the PA4-degrading enzyme gene

The open reading frame (ORF) encoding the PA4-degrading enzyme gene was identified from the draft genome of *Pseudoxanthomonas* sp. TN-N1, following the experimental method described in a previous study (Saito et al., 2023). Genomic DNA was extracted from *Pseudoxanthomonas* sp. TN-N1 using an Illustra Bacteria GenomicPrep Mini Spin Kit (Cytiva, Tokyo, Japan). Draft genome sequencing, genome assembly, and gene annotation were performed at Genewiz from Azenta Life Sciences (Japan). The purified enzyme was digested in-gel with trypsin, and peptide fragment sequences were analyzed using nano liquid chromatography-mass spectrometry (LTQ-Orbitrap XL mass spectrometer; Thermo Fisher Scientific, MA, United States). The ORF encoding the PA4-degrading enzyme was identified using a MASCOT search^1^ based on the peptide fragment sequences.

The enzyme gene was cloned into the pColdIV vector for expression in *E. coli*. The gene was amplified via PCR using primers pC-1_261-F (5′-AGGTAATACCATATGAAAGCGATACGCATGT TG-3′) and pC-1_261-R (5′-AGCAGAGATTACCTATCACGGA CACATCGGCGC-3′). The thermal cycling conditions used are: holding at 94 °C for 120 s, followed by 30 cycles of thermal denaturation at 94 °C for 15 s, annealing at 55 °C for 30 s, and extension at 68 °C for 156 s. Similarly, the pColdIV vector (TaKaRa Bio, Shiga, Japan) was PCR-amplified using primers pColdIV-F (5′-TAGGTAA TCTCTGCTTAAAAGC-3′) and pColdIV-R (5′-CATATGGTATT ACCTCTTAATAAT-3′) under the same cycling conditions, except for a final extension at 68 °C for 258 s. The plasmid for enzyme expression was constructed by an infusion reaction of both PCR products using 5 × In-fusion HD Enzyme Premix (TaKaRa Bio, Shiga, Japan) at 50 °C for 15 min. The resulting plasmid was named pColdIV-nyl4A*_px_*.

The enzyme gene was also cloned into the pNCMO2 vector for expression in *B. choshinensis*. For secretory expression of the PA4-degrading enzyme, the enzyme gene without the signal peptide sequence was inserted downstream of the signal peptide region of pNCMO2. The gene was amplified via PCR using primers pN-1_261-F (5′-CCCATGGCTTTCGCTCAGACCACCACGATCTTC AG-3′) and pN-1_261-R (5′-GGACACCAAATGGTGTCA CGGACACATCGGC GC-3′). The thermal cycling conditions used are: holding at 94 °C for 120 s, followed by 30 cycles of thermal denaturation at 98 °C for 10 s, annealing at 60 °C for 30 s, and extension at 68 °C for 75 s. Similarly, pNCMO2 (TaKaRa Bio, Shiga, Japan) was PCR-amplified using primers pNCMO2-F (5′-CACCATTTGG TGTCCAATTGC-3′) and pNCMO2-R (5′-AGCGAAAGCCATGGG AGCA-3′) under the same cycling conditions, except for a final extension at 68 °C for 157 s. The plasmid for enzyme expression was constructed via an infusion reaction of both PCR products using 5 × In-fusion HD Enzyme Premix at 50 °C for 15 min. The resulting plasmid was named pNCMO2-nyl4A*_px_*.

### 2.3 Heterologous expression of the PA4-degrading enzyme

*Escherichia coli* BL21(DE3) was transformed with pColdIV-nyl4A*_px_* or pColdIV according to the manufacturer’s protocol (Cosmo Bio, Tokyo, Japan). *E. coli* BL21(DE3) harboring pColdIV-nyl4A*_px_* or pColdIV were selected on LB plates containing 100 μg/mL ampicillin and then cultured in 5 mL of LB medium containing 100 μg/mL ampicillin at 30 °C overnight with shaking (160 strokes/min). The precultured solution was inoculated at a 1:100 ratio into 5 mL of LB medium containing 100 μg/mL ampicillin, and cultured at 30 °C with shaking (160 strokes/min). When the absorbance at 660 nm reached 0.5, the cultures were cooled at 15 °C for 30 min, after which isopropyl β-D-1-thiogalactopyranoside was added at a final concentration of 0.5 mM to induce gene expression. The cells were then cultured at 15 °C for 24 h with shaking (160 strokes/min).

*Brevibacillus choshinensis* HPD31-SP3 was transformed with pNCMO2-nyl4A*_px_* or pNCMO2 according to the manufacturer’s protocol (TaKaRa Bio, Shiga, Japan). *B. choshinensis* HPD31-SP3 harboring pNCMO2 or pNCMO2-nyl4A*_px_* were selected on MT plates containing 50 μg/mL neomycin and then cultured in 5 mL of TM medium containing 50 μg/mL neomycin at 30 °C overnight with shaking (160 strokes/min). The precultured solution was inoculated at a 1:100 ratio into 5 mL of TM medium containing 50 μg/mL neomycin, and cultured at 30 °C for 3 days with shaking (160 strokes/min).

After cultivation of the *E. coli* or *B. choshinensis* transformants, the cultures were centrifuged at 7,670 × *g* at 4 °C for 10 min, and the supernatants and bacterial cells were collected separately. The cells were suspended in 1 mL of 10 mM potassium phosphate buffer (pH 7.0) and crushed with 0.5-mm glass beads in a Multi-beads Shocker (Yasui Kikai, Osaka, Japan) in four cycles of 2,200 rpm for 120 s with 60 s intervals. Intracellular soluble and insoluble fractions were prepared by centrifuging the disrupted-cell suspension at 10,000 × *g* at 4 °C for 10 min. The intracellular insoluble fraction was resuspended in 1 mL of the same buffer. The culture supernatant and intracellular soluble and insoluble fractions were assayed for PA4-degrading activity and subjected to sodium dodecyl sulfate-polyacrylamide gel electrophoresis (SDS-PAGE) (Laemmli, 1970).

### 2.4 PA4-degrading activity assay

PA4 was synthesized and emulsified as previously described (Sasanami et al., 2022; Saito et al., 2023). The reaction mixture consisted of 100 μL of enzyme solution, 100 μL of the PA4 emulsion, and 100 μL of 1.5 M Tris–HCl buffer (pH 7.5). As a control, an enzyme solution denatured via heating at 100 °C for 10 min was used. The mixtures were incubated in 96-well plates at 30 °C with shaking. Degradation of PA4 was determined by measurement of the optical density at 655 nm using a microplate reader (Bio-Rad Laboratories, CA, United States). PA4-degrading activity was evaluated as the decrease in OD_655_ nm per hour.

### 2.5 Prediction of 3D structure and structural analysis

The 3D structure of the enzyme was predicted using ColabFold^2^ (Mirdita et al., 2022) based on the amino acid sequence without the signal peptide. Proteins with similar structures were identified by submitting the predicted 3D structures to the Dali server^3^ (Holm et al., 2023). Structural analyses and visualization were performed using ChimeraX v.1.5 (Meng et al., 2023).

### 2.6 BLAST analysis

To infer the function of Nyl4A*_px_*, BLASTP analysis (Altschul et al., 1997) was performed on the NCBI server. The full amino acid sequence of Nyl4A*_px_* was used as a query sequence. BLASTP search was conducted against the non-redundant sequence database using default parameters.

BLASTP analysis was also performed to estimate the environmental distribution of PA4-degrading enzymes. The catalytic domain regions of Nyl4A*_px_* and Nyl4A*_pa_* were used as query sequences. For metagenomic analyses, protein sequences derived from whole-genome shotgun metagenomic projects (env_nr) were used as the database. An *E*-value cutoff of 1E-10 was applied, and a sequence identity threshold of >30% was used as the criterion for protein homology. The data were actualized to July 9, 2025.

## 3 Results

### 3.1 Cloning and heterologous expression of the PA4-degrading enzyme gene

**Figure 1:**
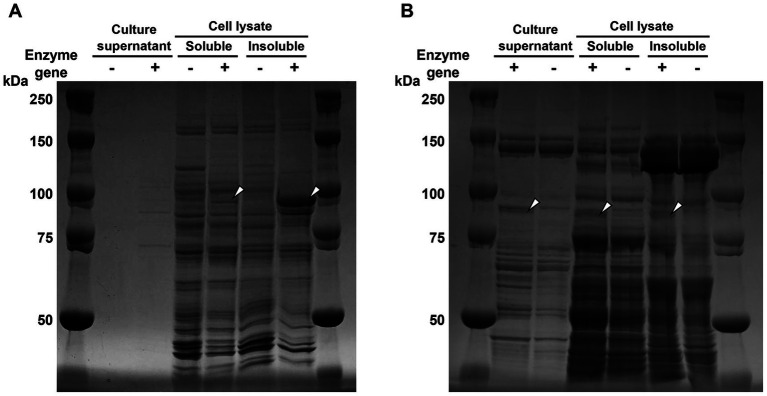
SDS-PAGE analysis of PA4-degrading enzyme expressed in recombinant *E. coli* BL21(DE3) and *B. choshinensis* HPD31-SP3. **(A)** PA4-degrading enzyme expressed in recombinant *E. coli* BL21(DE3). *E. coli* BL21(DE3) harboring pColdIV or pColdIV-nyl4A*_px_* were cultured in 5 mL of LB medium containing 100 μg/L ampicillin at 15 °C for 24 h with shaking (160 strokes/min). **(B)** PA4-degrading enzyme expressed in recombinant *B. choshinensis* HPD31-SP3. *B. choshinensis* HPD31-SP3 harboring pNCMO2 or pNCMO2-nyl4A*_px_* were cultured in 5 mL of TM medium containing 50 μg/mL neomycin at 30 °C for 3 days with shaking (160 strokes/min). White triangle indicates the bands derived from Nyl4A*_px_*.

The ORF predicted to encode the PA4-degrading enzyme gene was identified from 3,790 ORFs in the draft genome of *Pseudoxanthomonas* sp. TN-N1 using internal peptide fragment sequences of the purified PA4-degrading enzyme from this strain. The ORF was cloned into pColdIV and expressed in *E. coli* BL21(DE3). PA4-degrading activity was detected in the soluble fraction of cell lysate from *E. coli* harboring pColdIV-nyl4A*_px_* (Table 1). The PA4-degrading activity in *E. coli* BL21(DE3) reached 29.2 Δ655 nm/h/100 mL broth. SDS-PAGE analysis showed a protein band in the cell lysate fraction of *E. coli* harboring pColdIV-nyl4A*_px_* with a molecular weight consistent with the estimated molecular weight of the enzyme (89 kDa) based on its amino acid sequence (Figure 1A). These results indicate that the identified ORF encodes the PA4-degrading enzyme gene. However, most of the recombinant Nyl4A*_px_* accumulated in the insoluble fraction of the cell lysate. We hypothesized that intracellular expression of Nyl4A*_px_* in *E. coli* BL21(DE3) caused its insolubilization, as the enzyme is secreted by *Pseudoxanthomonas* sp. TN-N1. Therefore, for secretory expression of Nyl4A*_px_*, *B. choshinensis* HPD31-SP3, known for low extracellular protease activity and high protein synthesis and secretion capacity (Mizukami et al., 2015; Yao et al., 2020; Yao et al., 2022), was used as the host strain. When *B. choshinensis* HPD31-SP3 harboring pNCMO2-nyl4A*_px_* was cultivated for 3 days, PA4-degrading activity was detected in the culture supernatant (Table 1). The secreted Nyl4A*_px_* exhibited the PA4-degrading activity of 68.8 Δ655 nm/h/100 mL broth, which was higher than that observed for *E. coli* BL21(DE3). SDS-PAGE analysis revealed a band corresponding to Nyl4A*_px_* in the culture supernatant, indicating successful secretion of the enzyme by *B. choshinensis* HPD31-SP3 (Figure 1B).

**Table 1:** Heterologous expression of PA4-degrading enzyme by *E. coli* BL21(DE3) or *Brevibacillus choshinensis* HPD31-SP3.

Host	Plasmid	Fraction	Activity (Δ655 nm/h)	Enzyme production (Δ655 nm/h/100 mL broth)
*E. coli* BL21(DE3)	pColdIV-nyl4A*_px_*	Culture supernatant	0	0
		Cell lysate soluble	1.46 ± 0.121	29.2 ± 2.43
		Cell lysate insoluble	N. D.	N. D.
	pColdIV	Culture supernatant	0	0
		Cell lysate soluble	0.118 ± 0.075	2.37 ± 1.50
		Cell lysate insoluble	N. D.	N. D.
*B. choshinensis* HPD31-SP3	pNCMO2-nyl4A*_px_*	Culture supernatant	0.688 ± 0.090	68.8 ± 9.05
		Cell lysate soluble	1.30 ± 0.156	26.0 ± 3.12
		Cell lysate insoluble	N. D.	N. D.
	pNCMO2	Culture supernatant	0	0
		Cell lysate soluble	1.03 ± 0.132	20.6 ± 2.64
		Cell lysate insoluble	N. D.	N. D.

The data represent means ± SDs (*n* = 3).

N. D. indicates not detected. Although a slight decrease in absorbance was observed, clarification of the reaction mixture indicative of PA4 degradation was not detected after prolonged incubation.

### 3.2 Nucleotide sequence of the *nyl4A_px_* gene and prediction of its 3D structure

**Figure 2:**
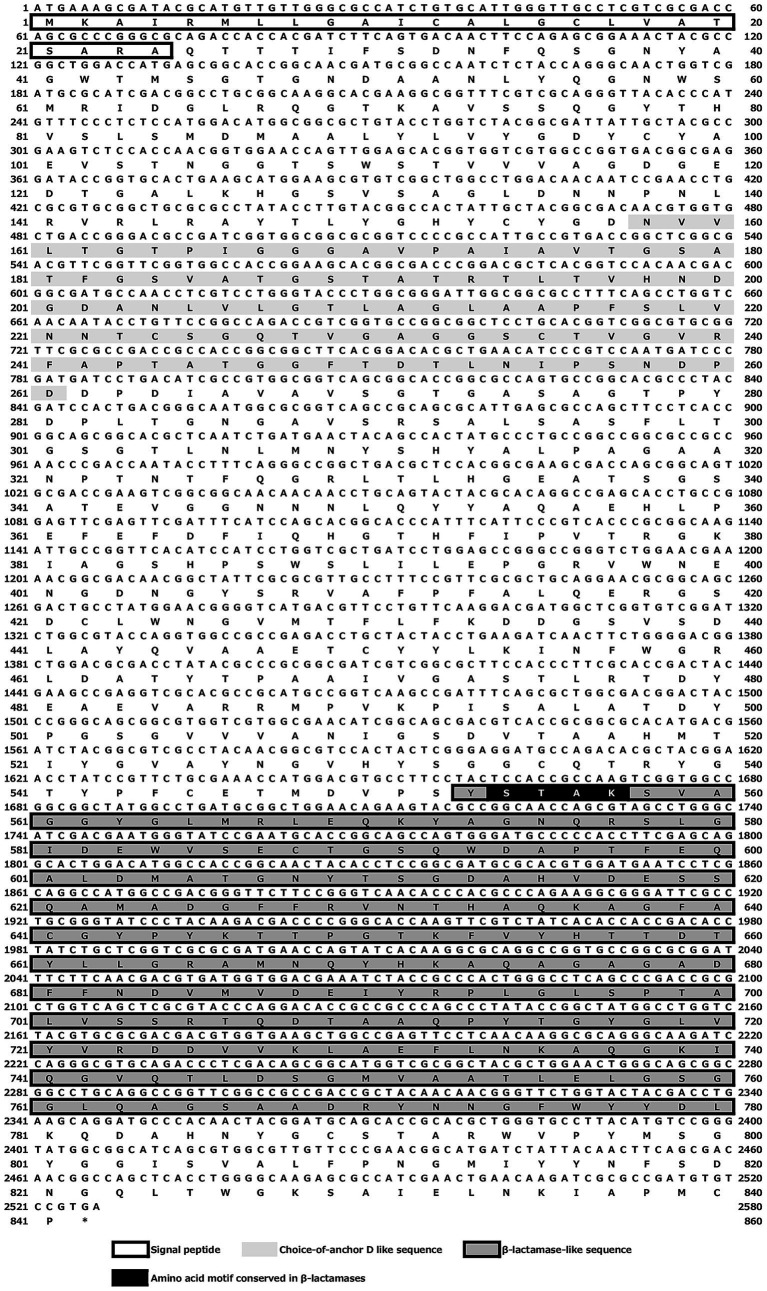
Nucleotide sequence of the PA4-degrading gene from *Pseudoxanthomonas* sp. TN-N1 and deduced amino acid sequence of the gene product (DDBJ Accession No. LC905122).

The *nyl4A_px_* gene (DDBJ Accession No. LC905122) consisted of 2,526 bp and encoded a protein of 841 amino acids with a calculated molecular weight of 89,006 Da (Figure 2). SignalP 6.0 analysis predicted that the N-terminal 24 amino acids of Nyl4A*_px_* constitute a sec-dependent signal peptide. A BLAST homology search against the non-redundant sequences using the full-length amino acid sequence showed that most of the hit sequences were uncharacterized proteins. Conserved domain search on the NCBI server revealed that the N158-D261 and Y553-L780 regions of Nyl4A*_px_* are homologous to choice-of-anchor D (Accession No. NF012200) and AmpC β-lactamase (Accession No. COG1680) (Jacoby, 2009), respectively (Figure 2). The Y553-L780 region contains the S-X-X-K motif (S554-K557) conserved in the β-lactamase family, and S554 was presumed to be the catalytic center.

**Figure 3:**
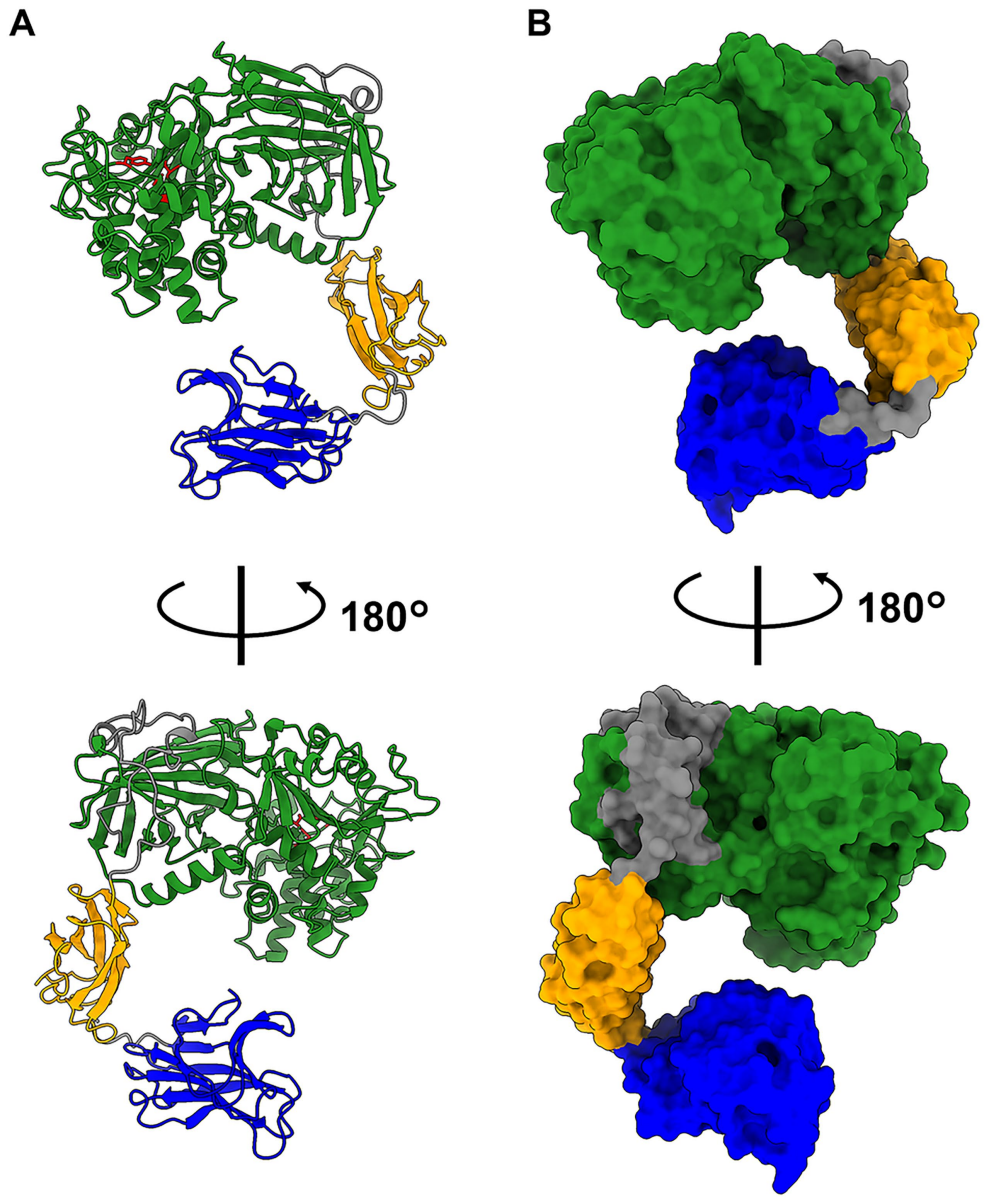
Predicted stereo views of the 3D structure of Nyl4A*_px_*. **(A)** Ribbon diagram of the monomer molecules of the enzyme. **(B)** Surface structure diagram of the monomer molecules of the enzyme. Residues in red indicate the three catalytic residues (S554/K557/Y655). Green, gray, yellow, and blue indicate the catalytic domain, linker domain, middle domain, and substrate-binding domain, respectively.

To infer the function of Nyl4A*_px_* at the structural level, the 3D structure of the enzyme was predicted using ColabFold based on the amino acid sequence without the signal peptide. The predicted subunit structure consisted of three structural domains: the N-terminal domain with a β-sandwich fold, the middle domain with a β-sandwich fold, and the C-terminal domain containing a β-barrel fold and a β-lactamase fold (Figure 3). To infer the functions of each domain in Nyl4A*_px_*, the predicted structures of each domain were subjected to the Dali server. The N-terminal domain showed structural similarity to carbohydrate-binding modules of polysaccharide-degrading enzymes [PDB ID: 2XOM (Cid et al., 2010), 4D0Q (Suits et al., 2014), and 4XUP (Sainz-Polo et al., 2015)]. The middle domain was structurally similar to immunoglobulin (Ig)-like folds in serine protease [PDB ID: 4LK4 (Gadwal et al., 2014), and 6BQM (Rule et al., 2020)]. The C-terminal domain showed structural similarity to β-lactamase family proteins [PDB ID: 7U1B (Daitch et al., 2023), and 8RLJ (Medrano et al., 2024)] and a 6-aminohexanoate-dimer hydrolase S112A/G181D/H266N mutant [PDB ID: 2DCF (Negoro et al., 2007)]. Based on the results of homology search using BLAST and Dali server, the N-terminal and C-terminal regions of Nyl4A*_px_* were predicted to function as the substrate-binding and catalytic domains, respectively.

### 3.3 Comparison of amino acid sequences and predicted 3D structures of Nyl4A*_pa_* and Nyl4A*_px_*

**Figure 4:**
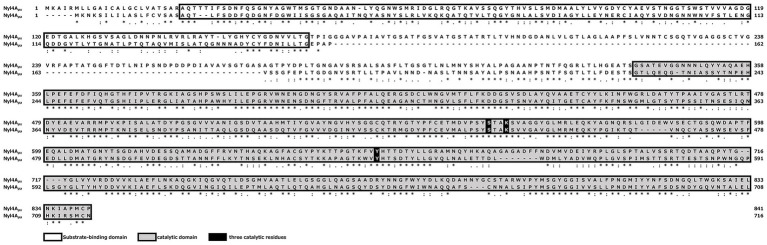
Amino acid sequence alignment between PA4-degrading enzymes from *Pseudoalteromonas* sp. Y-5 and *Pseudoxanthomonas* sp. TN-N1. Nyl4A*_pa_* and Nyl4A*_px_* indicate enzymes derived from *Pseudoalteromonas* sp. Y-5 and *Pseudoxanthomonas* sp. TN-N1, respectively. The alignment was constructed with ClustalW.

In the BLAST search using Nyl4A*_px_* as the query sequence, most of the hit sequences were uncharacterized proteins. Therefore, we compared the amino acid sequences and predicted 3D structures of Nyl4A*_px_* and Nyl4A*_pa_*, the only PA4-degrading enzyme whose gene has been identified to date (Saito et al., 2023). Nyl4A*_px_* shared 39% amino acid sequence identity with Nyl4A*_pa_* (Figure 4). Furthermore, the N-terminal region (A24-G163) of Nyl4A*_px_* exhibited 34% identity to the predicted substrate-binding domain of Nyl4A*_pa_*. The C-terminal region (G339-P841) of Nyl4A*_px_* also showed similarity to the predicted catalytic domain of Nyl4A*_pa_*, with an amino acid sequence identity of 48%. In addition, the three catalytic residues of Nyl4A*_pa_* (Ser, Lys, and Tyr) were conserved in the C-terminal region of Nyl4A*_px_*, suggesting that Ser554, Lys557, and Tyr655 were the catalytic residues of Nyl4A*_px_*. However, interestingly, Nyl4A*_pa_* lacked the amino acid sequence corresponding to the middle domain present in Nyl4A*_px_*.

**Figure 5:**
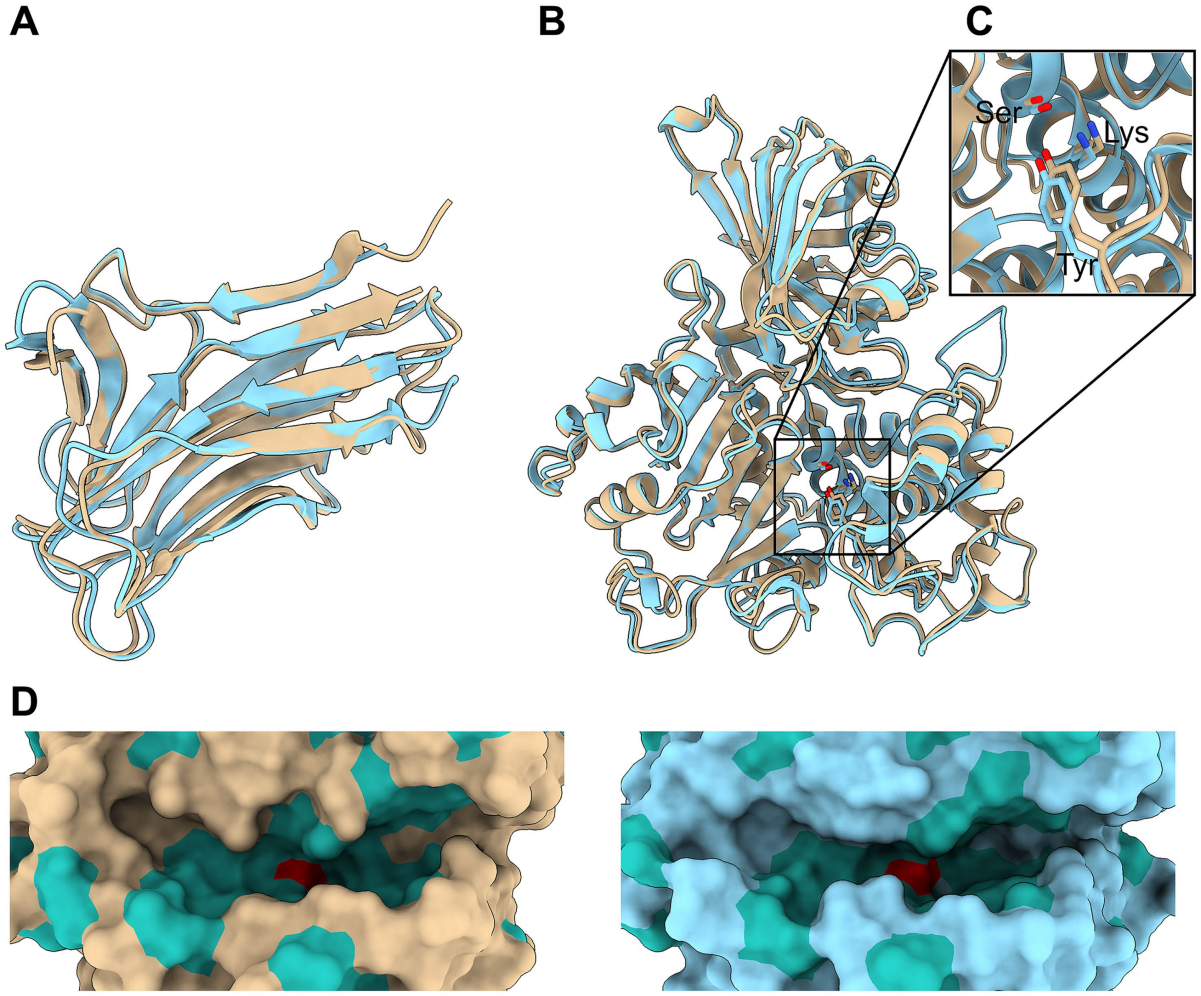
Superposition between PA4-degrading enzymes from *Pseudoalteromonas* sp. Y-5 and *Pseudoxanthomonas* sp. TN-N1. **(A)** Superposition between the substrate-binding domains. The predicted structures of both enzymes were overlapped using ChimeraX. Both enzymes overlapped with RMSD of 0.880 Å. Light blue and brown indicate Nyl4A*_pa_* and Nyl4A*_px_*, respectively. **(B)** Superposition between the catalytic domains. Both enzymes overlapped with RMSD of 0.786 Å. **(C)** Comparison of catalytic residues. **(D)** Surface structures of the catalytic clefts. Red and light sea green indicate the catalytic center and conserved amino acid residues in the two enzymes, respectively.

To compare the structures of Nyl4A*_px_* and Nyl4A*_pa_*, the predicted structures were overlapped using ChimeraX. The substrate-binding and catalytic domains of the two enzymes overlapped with root mean square deviations (RMSD) of 0.880 Å and 0.786 Å, respectively (Figure 5). Notably, the three catalytic residues (Ser, Lys, and Tyr) spatially overlapped in both enzymes (Figure 5C). In addition, while the amino acid residues at the ends and edges of the catalytic cleft differed, those surrounding the catalytic center were conserved in both enzymes (Figure 5D).

### 3.4 Distribution of Nyl4A*_px_* in the environment

Although Nyl4A*_px_* and Nyl4A*_pa_* were isolated from terrestrial and marine environments, respectively, both enzymes are sequentially and structurally similar. Therefore, we investigated whether PA4-degrading enzyme homologs are present in natural environments. To estimate the environmental distribution, BLASTP searches were performed against protein sequences derived from whole-genome shotgun metagenomic projects on the NCBI server. The catalytic domain region of Nyl4A*_px_* was used as the query sequence to identify homologs potentially possessing PA4-degrading activity. As a result, a total of 53 putative Nyl4A*_px_* homologs were detected from hydrothermal vent, marine, and marine sediment metagenomes (Table 2). Interestingly, no homologs were detected from terrestrial-associated metagenomes.

**Table 2:** BLAST hits using metagenomics database.

Queries	Environmental metagenomes	Score^a^	Number of hits^b^
Nyl4A*_px_*	Hydrothermal vent metagenome	509	4
	Marine metagenome	362	48
	Marine sediment metagenome	89.4	1
	Total		53
Nyl4A*_pa_*	Hydrothermal vent metagenome	538	4
	Marine metagenome	365	56
	Marine sediment metagenome	82.8	1
	Total		61

Proteins from whole-genome shotgun metagenomic projects (env_nr) were used as database.

^a^Score indicates the highest bit score for hits found from that metagenome.

^b^Number of hits indicates the total number of hits found that metagenome.

## 4 Discussion

Although several PA4-degrading microorganisms have been isolated from terrestrial environments (Yamano et al., 2008; Tachibana et al., 2010; Sasanami et al., 2022; Wang et al., 2023), only one PA4-degrading enzyme from a soil bacterium has been reported to date (Sasanami et al., 2022), and its gene has not yet been identified. In this study, we successfully identified the gene encoding the PA4-degrading enzyme from *Pseudoxanthomonas* sp. TN-N1, which was isolated from soil in our previous study. BLAST analysis revealed that Nyl4A*_px_* showed no sequence similarity to any previously characterized enzymes. However, Nyl4A*_px_* showed 39% amino acid sequence identity with Nyl4A*_pa_* (Figure 4), which we previously isolated from a marine environment (Saito et al., 2023). Among the domains of Nyl4A*_px_*, the predicted substrate-binding and catalytic domains exhibited sequence and structural similarities to those of Nyl4A*_pa_* (Figures 4, 5). The catalytic domains were more conserved than the substrate-binding domains of the two enzymes. Notably, the three catalytic residues spatially overlapped, and the amino acid residues surrounding the catalytic center were also conserved in both enzymes (Figures 5C,D). Our previous studies demonstrated that Nyl4A*_px_* and Nyl4A*_pa_* hydrolyze PA4 at amide bonds to produce GABA oligomers containing 2–4 mers and 2–3 mers, respectively (Sasanami et al., 2022; Saito et al., 2023). Together, these findings suggest that the catalytic domains of Nyl4A*_px_* and Nyl4A*_pa_* share a similar catalytic mechanism. In contrast, the amino acid residues at the ends and edges of the catalytic cleft differ in the two enzymes. These differences might explain why the chain lengths of PA4 degradation products differ in the two enzymes.

Based on homology searches and predicted structural features, Nyl4A*_px_* was found to contain the middle domain that is absent in Nyl4A*_pa_* (Figures 3, 4). Structural analysis using the Dali server suggested that this middle domain adopts an Ig-like fold, a structural motif widely distributed in both eukaryotes and prokaryotes. Ig-fold domains have been found in enzymes involved in diverse cellular processes, including biosynthesis, degradation, and transport, and are usually associated with binding functions (Bodelon et al., 2013). In particular, the carbohydrate-binding modules with Ig-fold have been reported to be involved in substrate-binding and structural stability (Hashimoto, 2006). Therefore, it is suggested that the middle domain of Nyl4A*_px_* could be involved in PA4 binding and structural stabilization of the enzyme. Elucidating the 3D structures of PA4-degrading enzymes is critical for understanding their structural properties and how structural differences affect their enzymatic properties.

To compare the mechanism of PA4 degradation by Nyl4A*_px_* and Nyl4A*_pa_* in more detail, it is necessary to purify both enzymes and investigate their enzymatic properties. Nyl4A*_pa_* from a marine environment has been purified at a yield of 822 Δ655 nm/h from *Pseudoalteromonas* sp. Y-5 (Saito et al., 2023), whereas Nyl4A*_px_* from a terrestrial environment has been purified at a yield of only 3.38 Δ655 nm/h from *Pseudoxanthomonas* sp. TN-N1 (Sasanami et al., 2022). In this study, we constructed heterologous expression systems for Nyl4A*_px_* using *E. coli* BL21(DE3) and *B. choshinensis* HPD31-SP3. Recombinant *B. choshinensis* HPD31-SP3 produced a larger amount of Nyl4A*_px_* than recombinant *E. coli* BL21(DE3). In *E. coli* BL21(DE3), Nyl4A*_px_* exhibited PA4-degrading activity of 29.2 Δ655 nm/h/100 mL broth; however, most of the enzyme accumulated in the insoluble fraction of the cell lysate (Table 1 and Figure 1A). In contrast, recombinant *B. choshinensis* HPD31-SP3 secreted Nyl4A*_px_* into the extracellular medium, and the PA4-degrading activity reached 68.8 Δ655 nm/h/100 mL broth (Table 1), representing a 2.4-fold increase compared with *E. coli* BL21(DE3). These results indicate that *B. choshinensis* HPD31-SP3 is a more suitable host for the production of PA4-degrading enzymes than *E. coli* BL21(DE3). *B. choshinensis* HPD31-SP3 has been widely used for heterologous protein production due to its non-pathogenic, low extracellular protease activity, and high secretory capacity (Mizukami et al., 2015; Yao et al., 2020; Yao et al., 2022). Therefore, we expected *B. choshinensis* HPD31-SP3 could become a platform for enzyme engineering of PA4-degrading enzymes. To purify recombinant Nyl4A*_px_*, a His-tag was added to the C-terminus of the enzyme. However, in *B. choshinensis* HPD31-SP3, the expression of the His-tagged enzyme was significantly reduced (data not shown). Optimization of expression conditions and tag design will be necessary for efficient production and purification of recombinant His-tagged Nyl4A*_px_* in future studies.

Finally, we investigated the environmental distribution of PA4-degrading enzymes by performing BLAST searches against metagenomic protein databases. Interestingly, although Nyl4A*_px_* was isolated from a soil bacterium, putative homologs of Nyl4A*_px_* were detected exclusively from marine-associated environmental metagenomes (hydrothermal vent, marine, and marine sediment metagenomes) (Table 2). A similar result was observed when the catalytic domain of marine-derived Nyl4A*_pa_* was used as the query sequence (Table 2). These results suggest that PA4-degrading enzymes similar to Nyl4A*_px_* and Nyl4A*_pa_* could be mainly distributed in marine environments. Notably, although PA4 is known to degrade well in soil, Nyl4A homologs are absent in terrestrial-associated metagenomes. This discrepancy suggests that other types of PA4-degrading enzymes, as yet unidentified, may be responsible for PA4 degradation in terrestrial environments. Further identification and characterization of soil-derived PA4-degrading enzymes will be essential for a comprehensive understanding of PA4 degradation mechanisms in terrestrial ecosystems.

## 5 Conclusion

In this study, we successfully identified the *nyl4A_px_* gene from *Pseudoxanthomonas* sp. TN-N1. BLAST search indicated that the amino acid sequence of Nyl4A*_px_* exhibits only limited similarity to that of previously characterized enzymes. However, the catalytic domain of Nyl4A*_px_* is structurally and sequentially similar to that of Nyl4A*_pa_* from a marine bacterium, suggesting that both enzymes share a similar PA4-degrading mechanism. These findings suggest that Nyl4A*_px_* and Nyl4A*_pa_* may be classified as members of a novel protein family. In addition, we constructed heterologous expression systems for Nyl4A*_px_* using *E. coli* BL21(DE3) and *B. choshinensis* HPD31-SP3. Compared with *E. coli* BL21(DE3), *B. choshinensis* HPD31-SP3 produced larger amounts of Nyl4A*_px_*. Therefore, *B. choshinensis* HPD31-SP3 is expected to become a promising platform for the enzyme engineering of PA4-degrading enzymes. Furthermore, BLAST searches against environmental metagenomes revealed putative homologs of Nyl4A*_px_* and Nyl4A*_pa_* in hydrothermal vent, marine, and marine sediment metagenomes, suggesting that PA4-degrading enzymes similar to these enzymes could be mainly distributed in marine environments. To our knowledge, this is the first report describing the structural properties of a PA4-degrading enzyme isolated from a soil bacterium. Overall, our findings provide new insights into the environmental distribution of PA4-degrading enzyme and contribute to a deeper understanding of the microbial degradation of PA4 in natural environments.

## Data Availability

The accession number for the enzyme gene presented in this paper can be found in the article.

## Footnotes

1
http://www.matrixscience.com/home.html

2
https://colab.research.google.com/github/sokrypton/ColabFold/blob/main/AlphaFold2.ipynb

3
http://ekhidna2.biocenter.helsinki.fi/dali/
